# Validation of the ND-PAE Diagnosis in Children with Heavy Prenatal Alcohol Exposure

**DOI:** 10.1007/s10578-024-01740-z

**Published:** 2024-07-31

**Authors:** Christina R. Veziris, Matthew T. Hyland, Julie A. Kable, Jeffrey R. Wozniak, Claire D. Coles, Philip A. May, Wendy O. Kalberg, Elizabeth R. Sowell, Kenneth L. Jones, Edward P. Riley, Sarah N. Mattson

**Affiliations:** 1 Center for Behavioral Teratology and Department of Psychology, San Diego State University, 6330 Alvarado Court, Suite 100, San Diego, CA 92120, USA; 2 Department of Psychiatry and Behavioral Sciences, Emory University School of Medicine, Atlanta, USA; 3 Department of Psychiatry and Behavioral Sciences, University of Minnesota, Minneapolis, USA; 4 Nutrition Research Institute, University of North Carolina, Chapel Hill, USA; 5 Center On Alcoholism, Substance Abuse, and Addiction, University of New Mexico, Albuquerque, USA; 6 Department of Pediatrics, Neurology, and Psychology, Keck School of Medicine of USC, Los Angeles, USA; 7 Department of Pediatrics, University of California San Diego School of Medicine, San Diego, USA

**Keywords:** Fetal alcohol spectrum disorders (FASD), Neurobehavioral disorder associated with prenatal alcohol exposure (ND-PAE), Prenatal alcohol exposure, Validity, Diagnosis

## Abstract

This study evaluated criteria for neurobehavioral disorder associated with prenatal alcohol exposure (ND-PAE). Kable et al. (Child Psychiatry Hum Dev 55:426, 2022) assessed the validity of this diagnosis in a sample with low exposure to alcohol. The current study expanded this assessment to a sample with a wider age range and heavier alcohol exposure. Data were collected from participants (5–17 years) with prenatal alcohol exposure (PAE) and typically developing controls at six Collaborative Initiative on Fetal Alcohol Spectrum Disorders sites using neuropsychological assessment and caregiver reports. Impairment was tested at 1SD, 1.5SD, and 2SD below the normative average and a modification of the adaptive functioning requirement was tested. Testing impairment at 1SD resulted in the highest endorsement rates in both groups. Our findings replicated the study by Kable et al. and show that current criteria captured a high rate of those with PAE and that requiring fewer adaptive functioning criteria resulted in higher sensitivity to PAE.

## Introduction

Prenatal alcohol exposure (PAE) may lead to fetal alcohol spectrum disorders (FASD) [1] which include fetal alcohol syndrome (FAS), partial FAS (PFAS), alcohol-related neurodevelopmental disorder (ARND), and alcohol-related birth defects (ARBD). As a group, these disorders are associated with neurobehavioral deficits, growth deficits, facial dysmorphology, and/or other medical symptoms [1]. Recent prevalence studies have suggested that FASD affects 1.1–5% of children in the U.S. [2], and it is one of the leading causes of birth defects and neurobehavioral deficits in children [3].

Despite these prevalence rates, diagnosis is particularly difficult due to confounding factors, such as incomplete or inaccurate self-report, lack of biomarkers, and comorbidities with other diagnoses (i.e., ADHD) [4], and delays in diagnosis can lead to an increased risk of adverse outcomes in childhood [5]. Diagnosis of FAS and PFAS is made, in part, based on facial dysmorphology [6] which can increase certainty of exposure and outcome. However, the majority of individuals with PAE or FASD do not manifest physical markers of their exposure [4], limiting the diagnostic utility of these features. Further, neurobehavioral challenges are present in individuals with PAE even in the absence of the characteristic physical features, and studies have shown that alcohol-exposed individuals with and without physical features have deficits in neuropsychological domains such as IQ, motor functioning, and learning [7, 8]. Neurobehavioral difficulties associated with FASD encompass cognition, behavior, and mental health. Cognitively, people with FASD experience deficits in general intelligence, executive functioning, attention, learning, memory, motor skills, and language [9]. FASD is associated with an elevated risk of mental health disorders, such as anxiety and depression [10, 11] as well as externalizing disorders such as attention-deficit/hyperactivity disorder, conduct disorder, and oppositional defiant disorder [10]. Some studies have shown that fewer dysmorphology features is associated with more behavioral problems [12]; however, other studies have shown that these symptoms occur at the same rate with and without physical features [13]. Therefore, creating a diagnosis that specifically reflects neurodevelopmental and mental health symptoms of PAE, rather than physical features, will increase early diagnosis and intervention, leading to fewer adverse outcomes.

In the Diagnostic and Statistical Manual of Mental Disorders, Fifth Edition (DSM-5), an FASD diagnosis termed neurobehavioral disorder associated with prenatal alcohol exposure (ND-PAE) was added under “Conditions for Further Study” to better detail the neurodevelopmental and mental health symptoms associated with PAE that are not captured with current FASD criteria [14–16]. In contrast to other diagnoses described under the FASD diagnostic umbrella which are medical diagnoses, ND-PAE is a mental health diagnosis and is not reliant on a dysmorphology evaluation [1, 16]. In contrast to the ARND diagnosis, the ND-PAE diagnosis, which is based on deficits in neurocognition, self-regulation, and adaptive functioning, is independent of facial dysmorphology and can be made in individuals with or without FAS or PFAS [1]. Like ARND, a diagnosis of ND-PAE requires confirmed PAE [1, 16]. The inclusion of the ND-PAE diagnosis in the DSM-5 broadens the expertise needed to diagnose individuals with PAE to include psychologists and other mental health professionals.

**Fig. 1:**
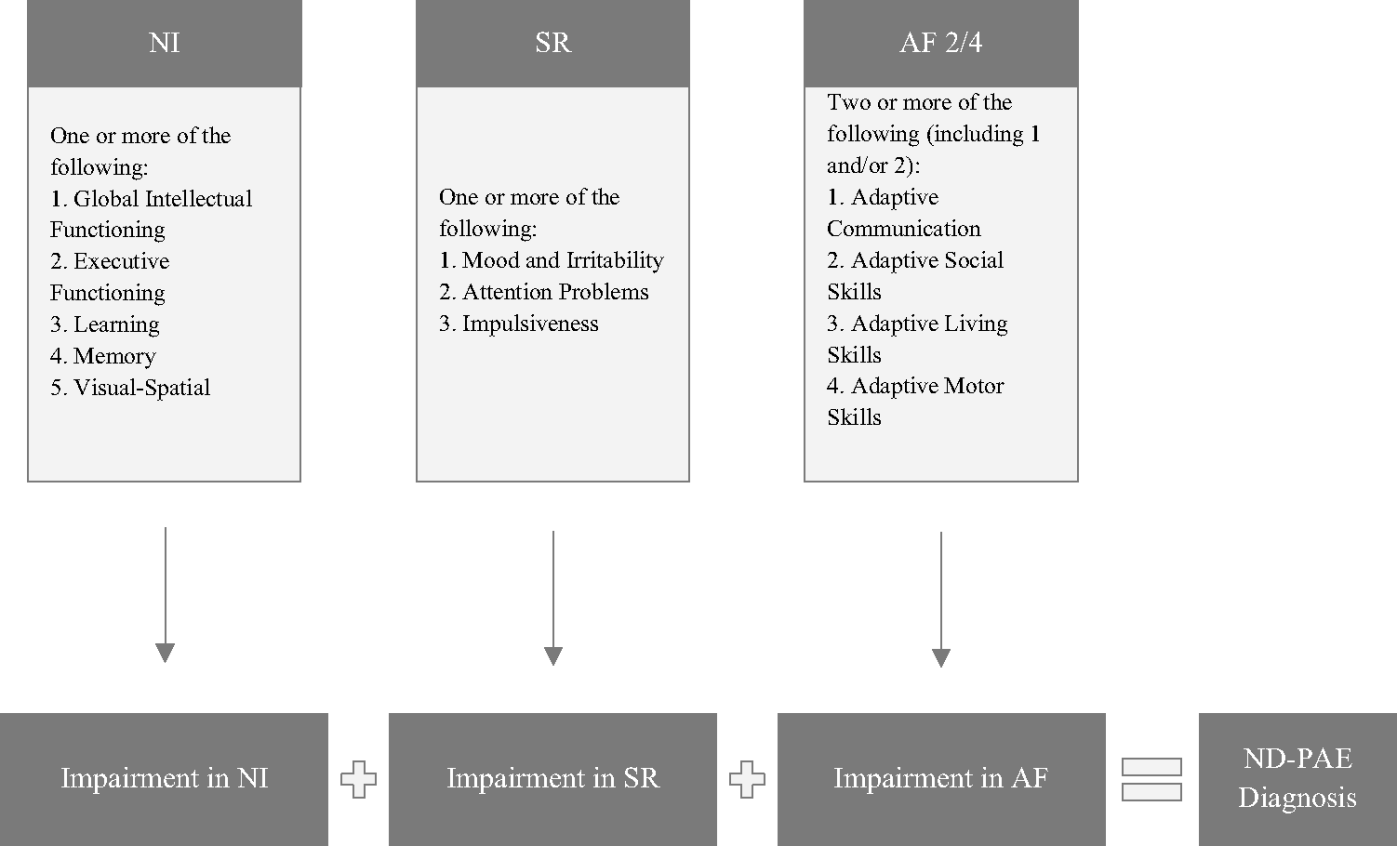
Requirements for the ND-PAE diagnosis as originally proposed. NI = neurocognitive, SR = self-regulation, and AF = adaptive functioning

ND-PAE criteria are categorized into three domains: neurocognitive, self-regulation, and adaptive functioning, and symptoms in all domains must first appear in childhood [14]. For neurocognitive impairment (NI), impairment must be present in one of the following categories: global intellectual functioning (which is represented by an IQ or parallel assessment of 70 or below), executive functioning, learning, memory, and visual spatial reasoning. For self-regulation (SR), impairment must be seen in mood or behavioral regulation, attention, and/or impulse control. Finally, in the adaptive functioning (AF) domain, deficits must be seen in two of the following categories: communication (i.e., acquiring and understanding language), social communication, daily living, and motor. At least one of the two deficits must be in communication or social communication. However, with the exception of global intellectual functioning (an IQ of 70 or below or a score of 70 or below on a similar comprehensive development assessment), the threshold for impairment in these domains is not clearly defined [17]. See Fig. 1 for the originally proposed ND-PAE criteria.

Previous studies have assessed the validity of the diagnosis and have found that the AF domain may be too restrictive [17]. Specifically, requiring two out of four criteria for the AF domain (AF 2/4) may not be necessary, and requiring that one of the two first criteria for adaptive functioning (communication and social communication) is not supported by the literature and therefore may lead to low sensitivity, or fewer true positives [18]. Sanders et al. [18] also evaluated construct and factorial validity of the ND-PAE diagnosis and found through principal component analysis (PCA) that symptoms only loaded on one factor, which included neurocognitive and adaptive functioning criteria. Their assessment of convergent and divergent validity showed that convergence, or the correlation among symptoms, was low among SR symptoms, specifically the correlation between mood regulation and attention symptoms [18]. Due to the unspecified impairment threshold in the DSM-5, previous studies have assessed symptom severity at 1.0, 1.5, and 2.0 standard deviations (SDs) from the mean [17, 18]. Internal validity analyses have shown that the symptoms have a fair internal consistency at both 1.0 and 1.5 SDs [18]. However, these studies have small sample sizes (n < 58).

A recent paper by Kable et al. [19] evaluated the ND-PAE diagnosis in a community sample of over 1000 1st-grade children (5–7 years) as a part of the Collaboration on FASD Prevalence (CoFASP) study. Participants were categorized as “at-risk” if they were exposed to more than minimal (> 13 drinks per month or 2 or more drinks on one occasion) amounts of alcohol prenatally or if they exhibited physical deficits such as facial dysmorphology or growth delays. This at-risk group was compared to a no risk group, categorized as not having any PAE and no related physical deficits (i.e., growth deficits and facial dysmorphology). They assessed criteria at both 1.0 and 1.5 SDs and assessed two different AF criteria for impairment: only one AF criterion needed (AF 1) or the original AF 2/4 criteria. Memory impairment for the NI domain was not assessed because it was not measured in all of the participants of this sample. Results showed higher endorsement of the ND-PAE diagnosis using 1.0 SD than 1.5 SD as the indicator of impairment and significant differences in ND-PAE endorsement between the at-risk and control groups at 1.0 SD. In the AF domain, having one necessary criterion (AF 1) led to a significant difference between the at-risk and no risk groups in AF endorsement at both 1.0 and 1.5 SDs, in contrast to the AF 2/4 criteria, which only had a significant difference between groups at the 1.0 SD cut-off. PCA was also performed to determine categories for the symptoms, which resulted in two factors: one with self-regulation, adaptive social skills, and adaptive independent living skills, and the other with IQ, visual-spatial functioning, adaptive communication, and adaptive motor skills. The authors used receiver operating characteristic (ROC) curves to test if each item and domain contributed to discrimination in ND-PAE endorsement (sensitivity and specificity). ROC curves produce area under the curve (AUC) values with values under 0.70 considered poor and values over 0.90 considered excellent. The results indicated that having an IQ score below 70 (i.e., 2.0 SD) did not significantly contribute to the diagnosis with either AF model in either of the groups (AUC < 0.60) and that the NI domain had poor prediction of the diagnosis with either AF model at 1.0 SD (AUC < 0.70). Therefore, they assessed IQ at 1.0 and 1.5 SDs and found that global intellectual impairment better predicted ND-PAE categorization at both 1.0 and 1.5 SD levels. Overall, the endorsement for the diagnosis was low among the at-risk group, which may be because these participants were recruited from the community instead of a healthcare facility and therefore may not be experiencing symptoms at the same rate or intensity as more heavily exposed individuals. Gender also seemed to play a role, as males had higher endorsement of symptoms than females in their sample.

Additional validation for this diagnosis is needed in a large, more heavily exposed sample. The purpose of the current study is to evaluate the ND-PAE criteria in a sample with heavier exposure and a wider age range.

## Methods

### Participants

Data were collected from participants with and without PAE at six sites (San Diego, Los Angeles, Northern Plains, Atlanta, New Mexico, and Minnesota) as part of an ongoing study for the Collaborative Initiative on Fetal Alcohol Spectrum Disorders (CIFASD), phases 2 through 4. Participants were categorized into two groups: an alcohol-exposed group (AE; *n* = 486) and a control group (CON; *n* = 679) with minimal or no PAE. General methodology for the CIFASD clinical projects has been described previously [see 20, 21].

Histories of PAE were collected from parent questionnaires as well as from medical, legal, and social service records [20] at each site. Participants were included in the AE group if they had confirmed reports of heavy (defined as an average of 14 or more standard alcoholic drinks per week or 4 or more drinks per occasion during pregnancy) or moderate (defined as an average of 1–13 drinks per week or 2–3 drinks per occasion during pregnancy) alcohol exposure. Participants were also included in the AE group if exposure was reasonably suspected based on documentation indicating significant problems related to alcohol use during pregnancy. Participants were included in the CON group if they had a confirmed lack of PAE or minimal exposure (defined as an average of < 1 drink per week and never more than 2 drinks per occasion during pregnancy) [20]. While recruitment varied slightly from phase to phase, all CIFASD phases included a heterogeneous control group that did not exclude participants based on other diagnosis (e.g., ADHD), clinical concerns or conditions, or receipt of special educational or social services [see 20, 22, 23]. Participants were excluded from analysis if they were missing data required for grouping (e.g., exposure information) or if they were entirely missing data for one or more of the domains.

### Neuropsychological Assessment

As part of the larger CIFASD study, children received a standardized neuropsychological battery administered by a trained examiner who was blind to exposure and diagnosis [20]. Parents or caregivers completed standardized questionnaires about medical and social history and child behavior. The test battery varied slightly depending on the phase of the CIFASD study, however all phases included measures of the domains required for the ND-PAE diagnosis: neurocognition, self-regulation, and adaptive functioning. Thus, not all participants had all measures, but all had at least one measure per domain, depending on criteria necessary for ND-PAE diagnosis (one criterion required for AF 1 versus two criteria required for AF 2/4). Criteria were assessed using neuropsychological testing measures and caregiver reports that best captured the symptoms of ND-PAE [24]. Individual measures were mapped to each domain required for the ND-PAE diagnosis, as detailed in Table 1.

#### Neurocognitive Domain

Neurocognitive impairment (NI) was captured using measures of global intellectual functioning, executive functioning, learning, visuospatial reasoning, and memory. For impairment in global intellectual functioning, the General Conceptual Ability (GCA) score from the Differential Ability Scales, Second Edition (DAS-II) [25] and the Full Scale IQ (FSIQ) score from the Wechsler Intelligence Scale for Children, Fourth and Fifth Edition (WISC-IV; WISC-V) [26, 27] were used. The original ND-PAE criteria specify that global intellectual functioning, captured by methods such as IQ, should be a standard score of 70 or below to be considered impaired (2.0 SD levels below the normative average). Following Kable et al. [19], IQ was also evaluated at 1.0 and 1.5 SD levels below the normative average. For the remaining NI criteria, impairment was assessed using measures from the Behavior Rating Inventory of Executive Function (BRIEF) [28], the Cambridge Neuropsychological Test Automated Battery (CANTAB) [29], the Child Behavior Checklist (CBCL) [30], the California Verbal Learning Test, Children’s version (CVLT-C) [31], the Delis-Kaplan Executive Function System (D-KEFS) [32], the Delis-Rating of Executive Function (D-REF) [33], NEPSY-II [34], the NIH Toolbox [35, 36], the Teacher Report Form (TRF) [30], the Wechsler Individual Achievement Test, Third Edition (WIAT-III) [37], and the WISC-IV/V.

#### Self-Regulation Domain

Self-regulation (SR) was captured using measures that assessed behavioral problems, and mood, attention, and impulsivity. Impairment for these symptoms was assessed using subtests from the Behavior Assessment System for Children, Third Edition (BASC-3) [38], the BRIEF, the CBCL, the Conners 3rd Edition (Conners 3) [39], the Disruptive Behavior Disorder questionnaire (DBD) [40], the NIH Toolbox, and the Vineland Adaptive Behavior Scales, Second and Third Editions (VABS-II; VABS-III) [41, 42].

#### Adaptive Functioning Domain

Adaptive functioning (AF) was captured using measures of communication, social skills, daily living skills, and motor functioning. Impairment in this domain was assessed using subtests from the BASC-3, CBCL, and VABS-II/III.

### Statistical Analysis

Each test was administered and scored according to standardized procedures provided by the test publisher. Prior to analysis, data were converted to z-scores to reflect standard deviations to put all data on the same scale (with the exception of the DBD, which only outputs a “1” for impairment or “0” for no impairment). Higher (positive) z-scores indicated stronger scores and lower (negative) z-scores indicated weaker scores. For example, IQ scores were converted from standard scores (M = 100; SD = 15) to z-scores (M = 0; SD = 1). All tests were corrected for age and/or sex, depending on the measure based on publisher-provided normative data.

Participants were each given scores for endorsement of the NI, SR, and AF domains at each SD level (i.e., 1.0, 1.5, and 2.0), as well as for the overall diagnosis with two AF criteria required (AF 2/4) or just one (AF 1). If the participant scored in the impairment range for any of the measures used for each criterion, they were coded as impaired for that criterion; if they did not score as impaired in any of the measures for a criterion, they were coded as “0”. Following the proposed ND-PAE diagnostic criteria, if a participant received a “1” in any of the criteria for the NI or SR domains, they were scored as *impaired* for that domain. For the AF 2/4 domain, participants had to be scored as “2” in the AF domain, with one of the criteria being either communication or social communication (AF criteria 1 and 2) deficit. For the AF 1 domain, participants were required to be scored as “1” in only one of the following AF criteria: impairment in communication, impairment in social communication, or impairment in daily living skills. Participants with impairment in the NI domain, SR domain, and one of the AF domains (AF 2/4 or AF 1) were categorized as ND-PAE. It is important to note that although we examined whether each individual met criteria for ND-PAE, in clinical settings the diagnosis of ND-PAE requires knowledge of PAE.

ROC curves were computed for each criterion and each domain at 1.0, 1.5, and 2.0 SD levels using SPSS v. 28.0.1.1. ROC analysis was also performed for ND-PAE risk at all three SD levels. Sensitivity, specificity, and accuracy results were calculated for ND-PAE endorsement at the 1.0, 1.5, and 2.0 SD levels to assess how well categorization was captured at each SD level. These calculations were also repeated at the 1.0 SD level using the original IQ criteria (2.0 SDs below the mean) to test if the original criteria best categorized the groups. Internal consistency among the symptoms was calculated using Cronbach’s alpha. Principal component analysis (PCA) was used to assess how factors load onto each domain at the different impairment levels (1.0, 1.5, and 2.0 SDs).

## Results

### Demographics

Participant demographics are listed in Table 2. Participants ranged from 5 to 17 years old. Participants were, on average, 10 years old with a slight majority of male participants in the AE group (*n* = 486, mean age = 10.9 years, female = 47%) and CON group (*n* = 679, mean age = 10.9 years, female = 43.6%). Average IQ was in the Low Average range for the AE group (mean IQ = 87.2, SD = 14.86) and in the Average range for the CON group (mean IQ = 94.4, SD = 16.98). Both groups included participants with ADHD (AE: 54.4%; CON: 24.5%).

There were no significant differences between mean ages (t(1128) = 0.058, p = 0.477, 95% CI [− 0.392, 0.416]) and sex (χ^2^(1) = 1.262, p = 0.261); however, the CON group had significantly higher IQ scores (t(1127) = − 12.683, p < 0.001, 95% CI [− 14.325, − 10.487]) and included fewer individuals diagnosed with ADHD (χ^2^(1) = 107.831, p < 0.001). The AE group included significantly more individuals that identified as white (χ^2^(1) = 5.397, p = 0.020) and significantly more individuals that identified as Hispanic/Latino (χ^2^(1) = 3.934, p = 0.047).

### ND-PAE Endorsement

See Table 3 for ND-PAE symptom and domain endorsement rates for each SD cut-off level. Using the original ND-PAE criteria with two of four AF criteria required (AF 2/4), 69.2% of participants from the AE group were categorized as ND-PAE (i.e., met criteria for ND-PAE excluding confirmed PAE) at the 1.0 SD level compared to 39.6% at the 1.5 SD level and 20.4% at the 2.0 SD level. When revising the AF criteria to only require one criterion (AF 1), 81.7% of the AE group were categorized as ND-PAE at the 1.0 SD level, compared to 52.5% at the 1.5 SD level and 32.4% at the 2.0 SD level. In the CON group, 22.6% of participants were identified as ND-PAE using AF 2/4 criteria at the 1.0 SD level, compared to 8.9% at the 1.5 SD level and 2.8% of participants at the 2.0 SD level. Using AF 1 criteria, 35.4% of the CON group were identified as ND-PAE at the 1.0 SD level, 15.2% at the 1.5 SD level, and 6.4% at the 2.0 SD level. There were significant differences between the AE and CON groups at each SD level in both the AF 2/4 model (1.0 SD: 69.2% vs. 22.6%, χ^2^(1) = 252.579, p < 0.001; 1.5 SD: 39.6% vs. 8.9%, χ^2^(1) = 157.173, p < 0.001; 2.0 SD: 20.4% vs. 2.8%, χ^2^(1) = 96.084, p < 0.001) and the AF 1 model (1.0 SD: 81.7% vs. 35.4%, χ^2^(1) = 245.466, p < 0.001; 1.5 SD: 52.5% vs. 15.2%, χ^2^(1) = 185.135, p < 0.001; 2.0 SD: 32.4% vs. 6.4%, χ^2^(1) = 134.362, p < 0.001).

For the NI domain, those in the AE group had significantly higher endorsement rates than the CON group at each SD level (1.0 SD: 97.8% vs. 82.7%, χ^2^(1) = 65.721, p < 0.001; 1.5 SD: 82.1% vs. 58.5%, χ^2^(1) = 73.086, p < 001; 2.0 SD: 67.3% vs. 40.0%, χ^2^(1) = 84.951, p < 0.001).

For the SR domain, those in the AE group had significantly higher endorsement rates than the CON group at each SD level (1.0 SD: 94.7% vs. 69.4%, χ^2^(1) = 112.808, p < 0.001; 1.5 SD: 85.0% vs. 48.2%, χ^2^(1) = 165.725, p < 0.001; 2.0 SD: 85.0% vs. 48.2%, χ^2^(1) = 165.725, p < 0.001).

For the AF domain, those in the AE group had significantly higher endorsement rates than the CON group at each SD level in the AF 2/4 model (1.0 SD: 70.8% vs. 25.5%, χ^2^(1) = 235.519, p < 0.001; 1.5 SD: 47.0% vs. 11.5%, χ^2^(1) = 183.554, p < 0.001; 2.0 SD: 26.4% vs. 4.5%, χ^2^(1) = 116.090, p < 0.001) and the AF 1 model (1.0 SD: 85.2% vs. 44.8%, χ^2^(1) = 195.638, p < 0.001; 1.5 SD: 64.9% vs. 23.3%, χ^2^(1) = 202.726, p < 0.001; 2.0 SD: 44.1% vs. 10.8%, χ^2^(1) = 168.993, p < 0.001).

A logistic regression was performed to analyze the relationship between age, sex, and ND-PAE risk. For this analysis, 35 participants (3%) were excluded because they were missing demographic data. At the 1.0 SD level, sex did not have a significant relationship with ND-PAE risk status in the AF 1 model (β = − 0.28, p = 0.815, 95% CI [0.768, 1.230]) or the AF 2/4 model (β = − 0.042, p = 0.729, 95% CI [0.755, 1.217]). Age was also not a significant predictor of ND-PAE risk status in the AF 1 model (β = 0.029, p = 106, 95% CI [0.994, 1.065]); however, it was a significant predictor in the AF 2/4 model (β = 0.036, p = 0.041, 95% CI [1.001, 1.074]). At the 1.5 SD level, age was a significant predictor in both models (AF 1: β = 0.042, p = 0.029, 95% CI [1.043, 1.082]; AF 2/4: β = 0.058, p = 007, 95% CI [1.016, 1.105]) but sex was not (AF 1: β = − 0.197, p = 0.133, 95% CI [0.636, 1.062]; AF 2/4: β = − 0.156, p = 0.287, 95% CI [0.642, 1.140]). At the 2.0 SD level, age was also a significant predictor in both models (AF 1: β = 0.083, p < 001, 95% CI [1.037, 1.138]; AF 2/4: β = 0.078, p = 0.008, 95% CI [1.020, 1.145]) and sex was a significant predictor of ND-PAE risk status in the AF 1 model (β = − 0.374, p = 0.022, 95% CI [0.499, 0.947]), but not in the AF 2/4 model (β = − 0.161, p = 0.418, 95% CI [0.576, 1.257]).

### Sensitivity, Specificity, and Accuracy Analyses

Specificity, sensitivity, and accuracy were calculated for both models at each SD level (see Table 4). The original ND-PAE criteria at a lowered impairment threshold produced the most accurate results (sensitivity: 69.1%, specificity: 77.5%, accuracy: 74.0%). However, the most sensitive outcome occurred using a lowered impairment threshold of 1.0 SD with the revised adaptive symptoms requirement (sensitivity: 81.7%), and the most specific outcome occurred using the original adaptive symptoms requirement and higher impairment threshold of 2.0 SD (specificity: 98.5%).

Although our analyses confirmed that the AF 2/4 model at the 1.0 SD level produced the most accurate categorization results, we tested whether this model was improved by using the original IQ criteria (2.0 SD levels below the mean), which increased specificity results while keeping all other criteria at the 1.0 SD level. Results of this analysis were comparable to the AF 2/4 model with the impairment level for all criteria at the 1.0 SD level (sensitivity: 68.9%; specificity: 77.5%; accuracy: 73.9%).

### Internal Consistency

Internal consistency among the ND-PAE criteria was assessed using the entire sample and then within each group (AE and CON) at each SD level. For the entire sample analysis, only 413 individuals were included in the analysis because they were not missing data for any of the ND-PAE criteria. Using the entire sample, Cronbach’s alpha was 0.833 at the 1.0 SD level, 0.831 at the 1.5 SD level, and 0.812 at the 2.0 SD level. In the AE group (*n* = 178), Cronbach’s alpha was 0.701 at the 1.0 SD level, 0.753 at the 1.5 SD level, and 0.766 at the 2.0 SD level. In the CON group (*n* = 233), Cronbach’s alpha was 0.772 at the 1.0 SD level, 0.764 at the 1.5 SD level, and 0.686 at the 2.0 SD level. This suggests that there is moderate to strong intercorrelation among the ND-PAE criteria at each SD level with the exception of the CON group at the 2.0 SD level [43].

### Principal Component Analysis

Principal component analysis (PCA) was performed using the 12 criteria at the 1.0 and 1.5 SD levels. Due to low accuracy levels from the sensitivity, specificity, and accuracy analyses, PCA was not performed for the 2.0 SD level as it was determined to not be a good cut-off for this diagnosis. A Promax rotation was used to allow for correlation among the symptoms. Factors were retained using Kaiser normalization and confirmed with parallel analysis [44]. Only 413 participants were included in this analysis because they had data for all criteria.

All SD levels had sufficient sampling adequacy for factor analysis (1.0 SD: Kaiser–Meyer–Olkin Measure of Sampling Adequacy (KMO) = 0.851, Bartlett’s Test of Sphericity χ^2^(66) = 1675.984, p < 0.001; 1.5 SD: KMO = 0.837, Bartlett’s Test of Sphericity χ^2^(66) = 1618.916, p < 0.001). For the 1.0 and 1.5 SD levels, three factors were derived and were weakly to moderately correlated at both SD levels (1.0 SD: factors 1 and 2: r = 0.513, factors 1 and 3: r = 0.122, factors 2 and 3: 0.241; 1.5 SD: factors 1 and 2: r = 0.471, factors 1 and 3: r = 0.302, factors 2 and 3: 0.367). Factor 1 had eigenvalues of 3.662 (explaining 37.4% of the variance) and 3.519 (explaining 36.0% of the variance) at the 1.0 and 1.5 SDs, respectively; factor 2 had eigenvalues of 3.528 (explaining 11.8% of the variance) and 3.299 (explaining 12.7% of the variance) at the 1.0 and 1.5 SDs, respectively; factor 3 had eigenvalues of 1.805 (explaining 8.7% of the variance) and 2.477 (explaining 10.4% of the variance) at the 1.0 and 1.5 SDs, respectively.

At the 1.0 SD level, items that loaded on factor 1 included impairment in executive functioning skills (NI domain), impairment of mood and behavioral regulation symptoms (SR domain), impairment in attention skills (SR domain), and impairment in impulse control (SR domain); items that loaded on factor 2 included impairment in global intellectual functioning (NI domain), impairment in adaptive communication (AF domain), social impairment (AF domain), impairment in independent living skills (AF domain), and adaptive motor functioning impairment (AF domain); factor 3 included learning impairments (NI domain), impairments in memory functioning (NI domain), and impairment in visual-spatial reasoning (NI domain). These results were also consistent at the 1.5 SD level with the exception of impairment in global intellectual functioning (NI domain), which shifted to factor 3.

### Receiver Operating Curve (ROC) Analysis

ROC analysis was used to assess the predictive validity of both domain (see AUC values in Table 5) and individual criteria endorsement (see AUC values in Table 6) in three different samples (i.e., overall sample, AE group only, CON group only) and using the original AF criteria requirements (AF 2/4 model) compared to using the modified AF requirements (AF 1 model). AUC values range from 0 to 1.0 with a chance level of 0.5. Values less than 0.70 indicate poor prediction, values between 0.70 and 0.80 indicate fair prediction, values between 0.80 and 0.90 indicate good prediction, and values higher than 0.90 indicate excellent prediction [45]. The impairment in adaptive motor skills symptom was not included in this analysis for the AF 1 model because it was not used as a criterion for impairment.

In the overall sample, each of the ND-PAE symptoms significantly contributed to the diagnosis in both models at all three SD levels. In the CON sample, all ND-PAE symptoms were significant predictors of diagnostic endorsement in both models at the 1.0 SD, but at the 1.5 SD and 2.0 SD levels, visual-spatial impairment was no longer a significant predictor for both models, and at the 2.0 SD level, global intellectual functioning also was no longer a significant predictor in the AF 2/4 model. In the AE group at the 1.0 SD level, all symptoms were significant predictors of being categorized as at-risk for ND-PAE with the exception of impairment in global intellectual functioning in the AF 2/4 model and impairment in memory and impairment in visual-spatial skills in both models. Visual-spatial impairment was also the only insignificant symptom at the 1.5 SD level in both models. At the 2.0 SD level in the AE group, all ND-PAE symptoms in the AF 2/4 model except for motor skills were significant predictors of diagnosis.

At the 1.0 SD level, the NI domain had poor (< 0.70) predictive validity for both models and for the overall sample, CON group, and AE group. However, the predictive validity becomes fair (≥ 0.70) for the overall sample and CON group at the 1.5 SD level, and it becomes fair to good (≥ 0.70 and < 0.90) for all three groups at the 2.0 SD level. For the SR domain, predictive validity was fair (≥ 0.70) at the 1.0 SD level for the overall sample and CON group for the AF 1 model only. At the 1.5 and 2.0 SD levels, the SR domain had fair to good predictive validity (≥ 0.70 and < 0.90) for only the overall sample and CON group for both models. For the AF domain, predictive validity was fair to excellent (≥ 0.70 and > 0.90) for both models and at all three SD levels.

AUC values for individual criteria in the NI domain varied from poor (< 0.70) to fair (≥ 0.70) for both models among the different samples and SD levels. Impairment in executive functioning was the only fair (≥ 0.70) predictor at all three SD levels but only in the overall sample and CON group and only within the AF 1 model. In addition, at the 2.0 SD level, impairment in executive functioning is also a fair predictor in the AE group in the AF 1 model and in the overall sample in the AF 2/4 model. At the 2.0 SD level, impairment in memory was also a fair predictor in the CON group only in the AF 2/4 model.

In the SR domain, both impairment in attention and impulse control had fair to good (≥ 0.70 and < 0.90) predictive validity at all three SD levels for the overall sample and CON group for both models, with the exception of the AF 2/4 model in the overall sample at the 2.0 SD level. Impairment in attention problems and impulsivity also had fair predictive validity in the AE group at the 1.0 SD level but only in the AF 1 model. Impairment in mood or behavioral regulation was a fair predictor (≥ 0.70) of ND-PAE risk for the overall sample and CON group for the AF 1 model at the 1.0, 1.5, and 2.0 SD levels, and at the 1.5 and 2.0 SD levels, it was also a fair predictor (≥ 0.70) for the overall sample and CON group in the AF 2/4 model.

In the AF domain, AUC values ranged from fair to excellent (≥ 0.70 and > 0.90) in all three samples, all three SD levels, and both models for the following criteria: impairment in adaptive communication, impairment in adaptive social skills, and impairment in adaptive living skills. The last AF criterion, impairment in motor skills, only had fair to good (≥ 0.70 and < 0.90) in the CON group in the AF 2/4 model at the 1.5 and 2.0 SD models.

ROC analysis was also used to assess how well domain impairment predicted group status (i.e., AE or CON). For the 1.0 SD level, AUC values were poor for the NI (AUC = 0.576, p < 0.001) and SR (AUC = 0.626, p < 0.001) domains but were fair for the AF 2/4 (AUC = 0.727, p < 0.001) and AF 1 (AUC = 0.702, p < 0.001) domains. For the 1.5 SD level, all domains were in the poor range (NI: AUC = 0.618, p < 0.001; SR: AUC = 0.684, p < 0.001; AF 2/4: AUC = 0.677, p < 0.001) with the exception of the AF 1 domain (AUC = 0.708, p < 0.001). At the 2.0 SD level, all domains were in the poor range (NI: AUC = 0.637, p < 0.001; SR: AUC = 0.684, p < 0.001; AF 2/4: AUC = 0.610, p < 0.001; AF 1: AUC = 0.666, p < 0.001).

## Discussion

This study aimed to validate the ND-PAE diagnosis currently located under the “Conditions for Further Study” section of the DSM-5. Using data from six CIFASD sites, individuals with moderate to heavy PAE were compared to individuals with minimal or no PAE and with and without other neurodevelopmental disorders on neuropsychological assessment measures that were tied to each ND-PAE criterion. Though the sample was smaller than the sample studied in Kable et al. [19], participants involved had experienced heavier exposure to alcohol prenatally and ranged in age and our sample was still large. Participants were of equal age and evenly divided in sex. Participants in the control group had higher IQ scores, and more participants in the AE group had IQ scores less than 85. ND-PAE endorsement was evaluated at 1.0, 1.5, and 2.0 SD levels, and a revision of the ND-PAE diagnosis (AF 1) was tested.

This study replicated a previous study done by Kable et al. [19] to validate the ND-PAE diagnosis in a sample with heavier exposure and a wider age range. Our findings replicated the study by Kable et al. [19] in two important ways: (1) current criteria captured a high rate of those with PAE and (2) requiring fewer adaptive functioning criteria was more sensitive to PAE. This higher rate of endorsement was reflected in both the alcohol-exposed group and the control group, again showing that an increase in capturing those at risk for ND-PAE will lead to an increase in false categorization of those not at risk (i.e., a sensitivity–specificity trade-off).

Our study also differed from Kable et al. [19] in a few important ways. Compared to the sample studied by Kable et al. [19], this sample showed higher ND-PAE endorsement rates of both the original (i.e., AF 2/4) and modified (i.e., AF 1) criteria in both the AE and CON groups at all three SD levels. Therefore, while these criteria better captured our heavily exposed sample, it also miscategorized more of our control group, especially seen in the NI and SR domains. However, there were significant differences found in endorsement rates of all criteria, domains, and diagnoses between the AE and the CON group, showing that the criteria better captured those with PAE than those without. This finding is in contrast to the study done by Kable et al. [19], which mainly found significant differences in endorsement rates between the at-risk and no risk groups at the 1.0 SD level. This difference may have occurred because our sample has heavier PAE than that of Kable et al. [19]. Also, though some domains may have high endorsement rates for the control group, the overall positive endorsement rates for the control groups were low, showing the necessity of requiring impairment in all three domains.

An increase in age was found to significantly increase the odds of being categorized as at-risk for ND-PAE in all models except the AF 1 model at the 1.0 SD level. This finding shows that older individuals are more likely to receive an ND-PAE diagnosis. This may be due to symptoms developing or worsening as individuals age, which emphasizes the importance of early diagnosis. Sex was only found to significantly impact the likelihood of being categorized as ND-PAE in the AF 1 model at the 2.0 SD level, with males being more likely to receive an ND-PAE diagnosis, but did not significantly impact the odds of being categorized as ND-PAE risk in any other models. This may be because our age group was larger than that of Kable et al. [19], and therefore the effects of sex on this diagnosis were diffused. However, because both age and sex have effects on ND-PAE diagnosis at different thresholds, future research should examine whether criteria for diagnosis should be adjusted based on age or gender.

Results from sensitivity, specificity, and accuracy analyses showed that the best classification outcomes occurred by using the original ND-PAE criteria (requiring two of the four AF criteria) and lowering the impairment threshold. Assessing IQ using the original criteria (i.e., 2.0 SD levels below the mean) and using modified criteria (i.e., 1.0 SD levels below the mean) had consistent results, showing that impairment levels for IQ have a low impact on overall accuracy in this heavily exposed sample. However, by using the revised ND-PAE criteria (requiring only one AF criterion) and a lowered impairment threshold, sensitivity is increased at the loss of specificity, and by using the original ND-PAE criteria and a higher impairment threshold, specificity is increased at the loss of sensitivity. Therefore, the criteria chosen will depend on the sensitivity and specificity balance desired, as increasing either would have very different impacts. For example, when screening for FASD, higher sensitivity may be desired so that more individuals are referred for a more comprehensive diagnostic evaluation.

Despite good internal consistency of the ND-PAE symptoms at each SD level and between and within groups, due to low accuracy rates, we found that the 2.0 SD was too high of an impairment threshold to accurately categorize for ND-PAE risk. Therefore, the 2.0 SD cut-off was excluded from the PCA. The results of our PCA showed that at all three SD levels, symptoms in the SR and AF domain were categorized together; however, the symptoms of the NI domain fit into different factors. This was different from the Kable et al. [19] study which found two-factor solutions for the 1.0 and 1.5 SD levels, loading NI and SR symptoms on their own factors with AF symptoms split between the two factors. Perhaps the heavier exposure level of our sample resulted in a better fit to the three domains laid out in the original criteria. Our PCA findings closely matched the original ND-PAE domains, with each domain loaded onto its own factor with the exception of executive function skills, which loaded with the SR symptoms, and global intellectual functioning, which loaded with the AF symptoms only at the 1.0 SD level; otherwise, it loaded with the NI symptoms. The change in factor loading for the global intellectual functioning symptom may be a function of increased impairment threshold. Greater impairment in global intellectual functioning may be more highly correlated with NI symptoms, but a lower severity in impairment may correlate more highly with AF symptoms.

Despite high endorsement rates at the 1.0 SD level, AUC values for the NI domain at the 1.0 SD level did not exceed chance levels, which was also seen in Kable et al. [19]. The only symptom of the NI domain that exceeded chance was impairment in executive functioning, which only exceeded chance in the overall and control samples. The original ND-PAE criteria suggests using an IQ score of 70 or below, but as seen in Kable et al. [19], this requirement was too restrictive. We tested IQ at all three SD levels below the normative average and found that this slightly improved predictive validity, which is congruent to findings by Kable et al. [19]. Visual-spatial impairment was found to not exceed chance in predicting ND-PAE risk in all samples at all three SD levels and was not a significant predictor of ND-PAE in the alcohol-exposed and control samples at varying SD levels. The AF domain had the highest AUC values at all three SD levels, which replicated Kable et al. [19] at the 1.0 and 1.5 SD levels. This shows the utility of the AF domain to differentiate both low- and heavy-exposed individuals from those without PAE. This finding occurred despite not including impairment in adaptive motor functioning as a criterion for the AF 1 model. We elected, a priori, not to include adaptive motor impairment as a sole indicator of adaptive functioning impairment. However, previous research supports motor impairment following PAE [4], and our PCA results show that this criterion fits with the other AF criteria. Therefore, future research can explore if this criterion can solely endorse the AF domain. The findings in the SR domain contrasted Kable et al. [19]. We found values exceeding chance in only two samples, the overall sample and control sample, at the 1.0 SD level, whereas Kable et al. [19] found values exceeding chance in all samples at that SD level. This may show that self-regulation impairment better differentiates those with lower PAE from those without PAE; however, it may also be due to the heterogeneity of our control group. Future analysis will need to be done to investigate if this may be the cause.

Limitations to this study include having a different set of neuropsychological assessment measures dependent on CIFASD phase. Importantly, all phases included measures of all the domains. This might be seen as a strength, however, as it validates that a variety of measures can be used for this diagnosis. In addition, some criteria were represented by more assessment measures than others due to assessment availability, which may increase the likelihood of receiving positive endorsement in a criterion. Multiple measures were included to confirm that each ND-PAE criterion was fully captured; however, though validated assessment measures were used, these analyses were done under the assumption that all measures equally assess each criterion. To eliminate this positive bias and differences due to measure variability, future research can focus on which measures best represent each criterion. Another limitation of this study was that though participants had measures reflective of at least one criterion in each domain, many participants were missing data for specific criteria, leading to a lower sample size for reliability analysis. We also had a heterogeneous control group that included individuals with minimal PAE and neurodevelopmental diagnoses such as ADHD. This may have increased endorsement rates in the CON group; however, individuals included in our control group with minimal PAE still would not have had a sufficient amount of PAE (i.e., more than 13 drinks per month or more than 2 drinks on any one occasion) to be included in the at-risk group studied in Kable et al. [19]. By including individuals with neurodevelopmental conditions in our control group, we are able to parse the effects of PAE from symptoms due to neurodevelopmental disorders. Also, this study used neuropsychological measures to identify impairment listed in the ND-PAE criteria which may not translate to most clinical settings.

Our findings support the utility of this diagnosis (with or without the AF modification) in a sample with heavy exposure and maintain many of the findings shown by Kable et al. [19]. However, adaptations to the original criteria may be helpful in order to truly capture ND-PAE risk. Though our results showed that the most accurate classification occurred at the lower impairment threshold (1.0 SD) using the original criteria (AF 2/4), sensitivity was increased when the AF criteria were modified (AF 1). Increasing sensitivity may be helpful in this population as it can increase earlier diagnosis and, in consequence, earlier treatment. Though the modified global intellectual functioning criterion tested (considering impairment as 1.0 or 1.5 SD levels below the normative average) did not exceed chance levels of prediction, it improved predictive validity, and therefore a lower impairment threshold for global intellectual functioning should be considered. Also, certain criteria, such as visual-spatial impairment and impairment in adaptive motor skills, did not exceed chance levels for prediction for ND-PAE risk, and therefore these criteria should be modified or excluded from the diagnosis.

By including this diagnosis in future versions of the DSM, children may receive earlier FASD diagnoses and earlier access to care. In addition, this diagnosis can expand the number of individuals with the expertise needed to diagnose and treat conditions associated with PAE. Therefore, adapting the criteria of this diagnosis to increase utility in diagnosing alcohol-exposed individuals may improve care in the FASD population.

## Summary

This study focuses on validating the Neurobehavioral Disorder associated with Prenatal Alcohol Exposure (ND-PAE). Recent studies have sought to validate the diagnosis, calling into question the degree of impairment required for the diagnosis. In this study, the proposed ND-PAE criteria were evaluated at 1.0, 1.5, and 2.0 standard deviations (SD) below the mean in a sample of children with heavy prenatal alcohol exposure (AE group) and typically developing children with and without other neurodevelopmental disorders (CON group). As in Kable et al. (2022), a revision of the adaptive functioning (AF) criteria was also evaluated. For endorsement of the ND-PAE diagnosis, our results indicated that using 1.0 SD below the mean was the most accurate in classifying those at-risk for ND-PAE (i.e., the AE group) from those not at-risk for ND-PAE (i.e., the CON group). For the global intellectual functioning criterion, there was no meaningful difference in specificity, sensitivity, or accuracy rates between 1.0 SD and 2.0 SD. The revised AF criteria did increase endorsement of the ND-PAE diagnosis in the AE group, and our results showed increased sensitivity of the diagnosis when revising these criteria. Our results also showed good internal consistency among the ND-PAE criteria and our PCA found a three-factor solution with symptoms in the self-regulation (SR) and AF domains categorized together by their respective domains and the neurocognitive (NI) symptoms assorted into the three factors. ROC analyses found that all criteria were significant predictors of ND-PAE endorsement across all SD levels with the exceptions of impairment in global intellectual functioning, impairment in memory, impairment in visual-spatial reasoning, and impairment in motor skills. Therefore, our findings support the proposed ND-PAE criteria with a revision in the global intellectual functioning criterion; however, revisions to the diagnosis will be dependent on the desired balance of sensitivity and specificity in clinical settings.

## Figures and Tables

**Table 1:** Measures used for each criterion

Criterion	Measures used

Neurocognitive Domain	
Impairment in Global Intellectual Performance	DAS-II GCA; WISC-IV/V FSIQ
Impairment in Executive Functioning	BRIEF Executive Composite Score; D-KEFS Verbal Fluency Switching Total Score, Trail Making Switching Scaled Score, Design Fluency Total Correct Scaled Score, Color-Word Interference Inhibition/Switching Scaled Score, Twenty Questions Total Initial Abstraction Scaled Score, Tower Test Total Achievement Scaled Score; D-REF Executive Functioning Score; NIH Toolbox Dimensional Change Card Sorting Subtest Score
Impairment in Learning	CBCL School Competence Scale Score; CVLT-C List A Total Trials 1–5 Total Correct Scaled Score, List A Trial 5 Free Recall Total Correct Z Score, Learning Slope Z Score, Percent Recall Consistency Z Score; TRF Academic Performance Scale Score and Learning Scale Score; WIAT-III Math Problem Solving and Word Reading Scores
Memory Impairment	CANTAB Working Memory Composite Score, Spatial Working Memory Score, Delayed Matching Score; CVLT-C Short Delay and Long Delay Free Recall Scores; NEPSY-II Narrative Memory Free Recall Score, Memory for Designs Total Scaled Score, Memory for Names Total Scaled Score; NIH Toolbox Picture Sequence Memory Subtest and List Sorting Working Memory Subtest Scores; WISC-IV/V Working Memory Composite Score
Impairment in Visual-Spatial Reasoning	CANTAB Spatial Working Memory Score; WISC-IV/V Block Design Scores
Self-Regulation Domain	
Impairment in Mood or Behavioral Regulation	BASC-3 Externalizing Problems and Internalizing Problems Scores; BRIEF Emotional Control Score; CBCL Anxious/Depressed and Withdrawn/Depressed Scores; VABS-II/III Externalizing and Internalizing Scores
Attention Deficit	BASC-3 Hyperactivity Score; CBCL Attention Problems Score; Conners 3 Inattention, Attention Deficit/Hyperactivity Disorder Predominantly Inattentive Type and Attention Deficit/ Hyperactivity Disorder Predominantly Hyperactive-Impulsive Type Scores; DBD ADHD Combined Diagnosis Score; NIH Toolbox Flanker Subtest Score
Impairment in Impulse Control	BASC-3 Aggression and Conduct Problems Scores; CBCL Rule Breaking Score; Conners 3 Hyperactivity/Impulsivity, Conduct Disorder, and Oppositional Defiant Disorder Scores; DBD ODD Diagnosis and CD Diagnosis Scores
Adaptive Functioning Domain	
Communication Deficit	BASC-3 Functional Communication Standard Score; VABS-II/III Communication Standard Score
Impairment in Social Communication	BASC-3 Social Skills Score; CBCL Social Problems Score; VABS-II/III Socialization Score
Impairment in Daily Living Skills	BASC-3 Act of Daily Living Score; VABS-II/III Daily Living Skills Standard Score
Impairment in Motor Skills	VABS-II/III Motor Skills Standard Score

**Table 2:** Demographics information for participants in the alcohol-exposed (AE) and control (CON) groups

Variable	AE (*n* = 486)	CON (*n* = 679)

Age at CBCL [M (SD)]	10.9 (3.32)	10.9 (3.45)
Sex [*n* (%) female]	228 (47.0%)	296 (43.6%)
Race [*n* (%) white]	188 (38.7%)	218 (32.2%)
Ethnicity [*n* (%) Hispanic/Latino]	109 (22.5%)	126 (18.6%)
FAS [*n* (%)]	71 (14.7%)	0 (0%)
ADHD Diagnosis [*n* (%)]	264 (54.4%)	166 (24.5%)
IQ^a^ [M (SD)]	87.2 (14.86)	94.4 (16.98)
IQ^a^ < 85 [*n* (%)]	207 (42.6%)	144 (21.3%)
Site		
San Diego [*n* (%)]	240 (49.4%)	240 (35.4%)
Atlanta [*n* (%)]	101 (20.8%)	207 (30.5%)
Los Angeles [*n* (%)]	34 (7%)	35 (5.2%)
Northern Plains [*n* (%)]	18 (3.8%)	22 (3.3%)
New Mexico [*n* (%)]	6 (1.3%)	22 (3.3%)
Minneapolis [*n* (%)]	87 (18%)	153 (22.6%)

a IQ is from test data, not based on parent report

**Table 3:** ND-PAE symptom and domain endorsement at each SD level

Domain	Specific symptom	AE group			CON group		
		% positive endorsement (1.0 SD)	% positive endorsement (1.5 SD)	% positive endorsement (2.0 SD)	% positive endorsement (1.0 SD)	% positive endorsement (1.5 SD)	% positive endorsement (2.0 SD)

Neurocognitive	Global intellectual functioning	42.6	23.9	12.0	21.3	9.5	3.9
Neurocognitive	Executive functioning	89.4	63.0	51.5	56	35	24.5
Neurocognitive	Impairment in learning	45.5	32.6	22.1	34.7	21.3	10.2
Neurocognitive	Impairment in visual-spatial reasoning	38.1	16.7	9.7	22.9	8.4	5.1
Neurocognitive	Memory impairment	67.1	46.1	30.7	53.1	30.8	18.6
Self-regulation	Impairment in mood and behavioral regulation	80.3	62.6	49.8	50.4	27.4	16.4
Self-regulation	Attention deficit	86.9	77.6	67.5	46.3	36.9	27.7
Self-regulation	Impairment in impulse control	77.8	68.2	62.0	42.9	32.5	26.9
Adaptive functioning	Adaptive communication deficit	68.8	44.1	24.3	27.0	13.7	5.5
Adaptive functioning	Adaptive social impairment	70.6	51.7	30.9	33.8	15.8	6.1
Adaptive functioning	Adaptive impairment in daily living	66.3	43.3	27.2	22.0	10.9	5.1
Adaptive functioning	Adaptive motor impairment	15.7	6.8	4.2	4.9	2.1	1.5
Overall domain and diagnostic endorsement							
Neurocognitive	1 Symptom	97.8	82.1	67.3	82.7	58.5	40.0
Self-regulation	1 Symptom	94.7	85.0	85.0	69.4	48.2	48.2
Adaptive functioning	2 of 4 Symptoms	70.8	47.0	26.4	25.5	11.5	4.5
ND-PAE diagnosis	4 Symptoms (Neurocognitive, Self-Regulation, AF 2 of 4)	69.2	39.6	20.4	22.6	8.9	2.8
Modified AF criteria							
Adaptive functioning	1 Symptom	85.2	64.9	44.1	44.8	23.3	10.8
ND-PAE diagnosis	3 Symptoms (Neurocognitive, Self-Regulation, AF 1)	81.7	52.5	32.4	35.4	15.2	6.4

**Table 4:** Sensitivity, specificity,and accuracy analyses

	1.0 SD		1.5 SD		2.0 SD		2.0 SD (IQ only) + 1.0 SD
	AF 1 (%)	AF 2/4 (%)	AF 1 (%)	AF 2/4 (%)	AF 1 (%)	AF 2/4 (%)	AF 2/4 (%)

Sensitivity	81.7	69.1	52.5	39.5	32.3	20.4	68.9
Specificity	64.7	77.5	84.8	91.2	96.7	98.5	77.5
Accuracy	71.8	74.0	71.3	69.6	61.4	55.7	73.9

**Table 5:** AUC values for eachdomain for each SD level

Group	Model	1.0 SD Threshold cut-off level			1.5 SD Threshold cut-off level			2.0 SD Threshold cut-off level		
		NI	SR	AF	NI	SR	AF	NI	SR	AF

Overall sample	AF 1	**0.622**	**0.722**	**0.923**	**0.729**	**0.763**	**0.929**	**0.794**	**0.720**	**0.955**
	AF 2/4	**0.595**	**0.673**	**0.979**	**0.702**	**0.733**	**0.970**	**0.771**	**0.703**	**0.981**
CON group	AF 1	**0.634**	**0.737**	**0.927**	**0.745**	**0.806**	**0.952**	**0.821**	**0.777**	**0.976**
	AF 2/4	**0.612**	**0.698**	**0.981**	**0.728**	**0.784**	**0.985**	**0.809**	**0.767**	**0.992**
AE group	AF 1	0.562	**0.646**	**0.904**	**0.688**	**0.658**	**0.870**	**0.742**	**0.611**	**0.913**
	AF 2/4	0.537	**0.587**	**0.973**	**0.648**	**0.624**	**0.939**	**0.705**	**0.594**	**0.963**

Significant values are given in bold font

**Table 6:** AUC values for each criterion for each SD level

Group	Model	1.0 SD Threshold cut-off level											
		Global intellectual functioning	Executive functioning	Learning	Memory	Visual-spatial	Mood and irritability	Attention problems	Impulsiveness	Adaptive communication	Adaptive social skills	Adaptive living skills	Adaptive motor skills

Overall sample	AF 1	**0.649**	**0.726**	**0.590**	**0.617**	**0.570**	**0.723**	**0.799**	**0.765**	**0.851**	**0.848**	**0.825**	–
	AF 2/4	**0.652**	**0.696**	**0.588**	**0.599**	**0.557**	**0.699**	**0.766**	**0.734**	**0.888**	**0.857**	**0.884**	**0.693**
CON group	AF 1	**0.631**	**0.708**	**0.571**	**0.630**	**0.560**	**0.710**	**0.780**	**0.735**	**0.810**	**0.826**	**0.752**	–
	AF 2/4	**0.658**	**0.678**	**0.575**	**0.630**	**0.568**	**0.685**	**0.779**	**0.721**	**0.886**	**0.852**	**0.859**	**0.668**
AE group	AF 1	**0.601**	**0.655**	**0.608**	0.561	0.490	**0.668**	**0.722**	**0.735**	**0.856**	**0.856**	**0.851**	–
	AF 2/4	0.585	**0.620**	**0.585**	0.533	0.504	**0.642**	**0.651**	**0.667**	**0.854**	**0.842**	**0.856**	**0.697**

Group	Model	1.5 SD threshold cut-off level											
		Global intellectual functioning	Executive functioning	Learning	Memory	Visual-spatial	Mood and irritability	Attention problems	Impulsiveness	Adaptive communication	Adaptive social skills	Adaptive living skills	Adaptive motor skills

Overall sample	AF 1	**0.630**	**0.730**	**0.642**	**0.672**	**0.545**	**0.751**	**0.783**	**0.767**	**0.821**	**0.837**	**0.786**	–
	AF 2/4	**0.656**	**0.686**	**0.653**	**0.664**	**0.544**	**0.730**	**0.767**	**0.735**	**0.875**	**0.889**	**0.870**	**0.658**
CON group	AF 1	**0.607**	**0.729**	**0.644**	**0.673**	0.519	**0.756**	**0.821**	**0.801**	**0.808**	**0.825**	**0.744**	–
	AF 2/4	**0.633**	**0.665**	**0.685**	**0.669**	0.490	**0.743**	**0.819**	**0.752**	**0.881**	**0.907**	**0.878**	**0.795**
AE group	AF 1	**0.621**	**0.683**	**0.638**	**0.660**	0.537	**0.675**	**0.670**	**0.675**	**0.803**	**0.798**	**0.767**	–
	AF 2/4	**0.645**	**0.634**	**0.629**	**0.642**	0.546	**0.646**	**0.651**	**0.647**	**0.845**	**0.835**	**0.830**	**0.625**

Group	Model	2.0 SD threshold cut-off level											
		Global intellectual functioning	Executive functioning	Learning	Memory	Visual-spatial	Mood and irritability	Attention problems	Impulsiveness	Adaptive communication	Adaptive social skills	Adaptive living skills	Adaptive motor skills

Overall sample	AF 1	**0.612**	**0.752**	**0.652**	**0.654**	**0.555**	**0.719**	**0.755**	**0.735**	**0.794**	**0.821**	**0.753**	–
	AF 2/4	**0.612**	**0.735**	**0.686**	**0.649**	**0.570**	**0.709**	**0.753**	**0.698**	**0.878**	**0.890**	**0.868**	**0.629**
CON group	AF 1	**0.619**	**0.742**	**0.657**	**0.686**	0.510	**0.711**	**0.787**	**0.779**	**0.744**	**0.803**	**0.721**	–
	AF 2/4	0.537	**0.672**	**0.691**	**0.729**	0.501	**0.768**	**0.791**	**0.768**	**0.851**	**0.929**	**0.853**	**0.861**
AE group	AF 1	**0.600**	**0.708**	**0.636**	**0.628**	**0.565**	**0.645**	**0.651**	**0.640**	**0.800**	**0.799**	**0.733**	–
	AF 2/4	**0.614**	**0.691**	**0.668**	**0.608**	**0.577**	**0.612**	**0.647**	**0.593**	**0.867**	**0.845**	**0.842**	0.600

Significant values are indicated by bold font

## Data Availability

All or part of this work was done in conjunction with the Collaborative Initiative on Fetal Alcohol Spectrum Disorders (CIFASD), which is funded by grants from the National Institute on Alcohol Abuse and Alcoholism (NIAAA). Additional information about CIFASD, including about data-sharing opportunities, can be found at www.cifasd.org.
